# Vestibular Neuritis Following COVID-19 Vaccination: A Retrospective Study

**DOI:** 10.7759/cureus.24277

**Published:** 2022-04-19

**Authors:** Teru Kamogashira, Hideaki Funayama, Shinnosuke Asakura, Shinichi Ishimoto

**Affiliations:** 1 Department of Otolaryngology, JR Tokyo General Hospital, Tokyo, JPN; 2 Department of Otolaryngology and Head and Neck Surgery, University of Tokyo, Tokyo, JPN; 3 Department of Clinical Examination, JR Tokyo General Hospital, Tokyo, JPN

**Keywords:** bnt162b2, vertigo, vaccination, covid-19, vestibular neuritis

## Abstract

Objective

To determine if the COVID-19 vaccine can cause vestibular neuritis (VN).

Design

Retrospective study.

Setting

Vertigo outpatient clinic of the Department of Otolaryngology JR Tokyo General Hospital.

Participants:

378 patients who presented at the Vertigo clinic between July 2018 and March 2022

Results

23 out of 378 cases were diagnosed with vestibular neuritis. There was a significant seasonal bias of the onset of VN in 2021-3Q compared to other seasons. All 7 patients diagnosed with VN whose onset was 2021-3Q and 2021-4Q had received the BNT162b2 (Pfizer-BioNTech) vaccine within the previous 3 months and one patient diagnosed with VN whose onset was 2022-1Q had a history of COVID-19 infection six months earlier.

Conclusions

VN should be recognized as one of the side-effects of the BNT162b2 COVID-19 vaccination.

## Introduction

The outbreak of atypical viral pneumonia in Wuhan, Hubei Province, China, later named coronavirus disease 2019 (COVID-19), in December 2019 was found to be caused by a new coronavirus strain named severe acute respiratory syndrome coronavirus 2 (SARS-CoV-2) [1,2]. COVID-19 has spread to countries around the world and affected hundreds of millions of people since it was declared a public health emergency of international concern by the World Health Organization (WHO) in March 2020 [3]. Several types of vaccines have been developed to date against COVID-19 with the aim of controlling the pandemic and its socioeconomic impact [4], including Tozinameran (BioNTech-Pfizer, Comirnaty, BNT162b2) [5], Elasomeran (Moderna, Spikevax, mRNA-1273) [6], ChAdOx1 nCoV19 (AstraZeneca-Oxford University, Vaxzevria, AZD1222) [7], and Ad26.COV2.S (Janssen, COVID-19 Vaccine Janssen) [8]. Although the COVID-19 vaccine has been successful in reducing the rate of infection, the severity of illness, hospitalization, and mortality in various populations during the early stages of the epidemic [9], the occurrence and spread of mutant strains of COVID-19 continue alongside the widespread use of the vaccine, and hence the evaluation of the efficacy and safety of multiple doses of the vaccine in the future is critical.

In assessing efficacy and safety, it is important to accurately evaluate the rates of comorbidities associated with infection and any adverse effects associated with the vaccination. The comorbidities associated with COVID-19 infection in the otorhinolaryngological field have included sudden hearing loss [10], vestibular neuritis (VN) [11], and dizziness [12]. Most of the otological problems associated with COVID-19 infection have been reported to improve within a few weeks and are generally not long-term [13]. The common otorhinolaryngological disorders reported after COVID-19 vaccinations have been tinnitus [14] and facial nerve palsy [15]. Facial nerve palsy is thought to be caused by the reactivation of the herpes simplex virus (HSV) or varicella-zoster virus (VZV) [16], and the reactivation of VZV after COVID-19 vaccination has been reported [17], along with many reports of diseases associated with VZV reactivation after COVID-19 vaccination in the fields of dermatology and ophthalmology [18]. VN is considered to be associated with HSV reactivation [19,20], and is presumed to be one of the side effects of the vaccine, because the vestibular nerve passes through the internal auditory canal, as does the facial nerve; however, the reported cases have been scarce [21,22]. The purpose of this study is to assess the possibility of VN after COVID-19 vaccination via a retrospective analysis of cases in a vertigo outpatient clinic.

## Materials and methods

This was a retrospective study conducted at a single institution. Patients referred to the vertigo outpatient clinic, Department of Otolaryngology at the JR Tokyo General Hospital between July 2018 and March 2022 were included in the study. Data from 2018 was included to help observe any seasonal bias. The study was approved by the regional ethical standards committee of the JR Tokyo General Hospital (R02-03). Caloric testing was performed in a darkened room by irrigating the external auditory canal with 20-mL ice water (4 °C) for 10 seconds. Caloric nystagmus was recorded using videonystagmography (Interacoustics A/S, Middelfart, Denmark). An abnormal caloric response was defined based on either of the following criteria: (1) canal paresis (CP) percentage ≥20% [23] or (2) maximum slow phase eye velocity <10 degree/s [24]. The cervical vestibular evoked myogenic potential (cVEMP) and ocular VEMP (oVEMP) were recorded with the Nicolet EDX system (Natus Medical Incorporated, San Carlos, CA) by using a tone burst stimulus of 500 Hz at 125 dBpeSPL (rise, 1 ms; plateau, 2 ms; and fall, 1 ms) and a tone burst of 1kHz at 125 dBpeSPL (rise, 1 ms; plateau, 2 ms; and fall, 1 ms). In all patients, the following criteria were applied for VN diagnosis [25]: (1) a sudden attack of vertigo, (2) a persistent feeling of lightheadedness during physical movement or walking following the vertigo attack, (3) no auditory symptoms associated with vertigo, such as hearing loss, tinnitus, or ear blockage, (4) no neurological symptoms other than those of the 8th cranial nerve, (5) unilateral or bilateral peripheral vestibular dysfunction (semicircular canal hypofunction), identified by an abnormal caloric response, (6) fixed horizontal or mixed horizontal gyration nystagmus revealed by spontaneous and positional nystagmus testing during attacks of vertigo, (7) normal hearing or hearing loss unrelated to vertigo, and (8) exclusion of known causes, such as inner ear or posterior labyrinthine disease, cerebellar or central brainstem disease, which may cause vertigo symptoms similar to VN. Microsoft Excel 2010 was used for processing data. The Smirnov-Grubbs test was used to detect outliers by using the R software version 4.1.1 (The R Foundation for Statistical Computing, Vienna, Austria, 2021).

## Results

A total of 378 patients [166 males and 212 females; median age: 61 years (interquartile range: 45-73 years; range: 19-91 years)] were examined in the vertigo outpatient clinic between 2018-3Q and 2022-1Q, and 23 patients [10 males and 13 females; median age: 64 years (interquartile range: 50-78 years; range: 31-85 years)] were diagnosed as having VN. For each quarter, the total number of examinations, the number of VN cases based on the date of examination, the rate of VN cases per number of examinations, and the number of cases of VN based on the date of onset are shown in Table 1. The onset of two cases had been five and 20 years before the examination date, and hence they were excluded from the onset date table. The total number of examinations in 2020-2Q fell sharply because the COVID-19 lockdown was enforced during this period. The vaccination program in the prefecture where the hospital in this study is located was launched in 2021-Q2. There was a significantly increased incidence of VN cases in 2021-3Q (Table 1). All seven patients diagnosed with VN whose onset was 2021-3Q or 2021-4Q had received BNT162b2 in the preceding three months. One patient diagnosed with VN whose onset was 2022-1Q had a history of COVID-19 infection six months earlier, while the other cases did not have a history of COVID-19 infection at the time of the examination date. The results of the vestibular function tests in these seven patients are shown in Table 2. The common comorbidities were hypertension and hyperlipidemia. All seven patients had no colds or upper respiratory tract infections prior to the onset of VN. The CP percentage on caloric testing was 100% in many cases, suggesting severe peripheral vestibular dysfunction. The affected side evaluated with VEMP testing did not match the affected side evaluated with caloric testing in some cases. All patients are undergoing continuous rehabilitation at the time of writing this report.

**Table 1:** Number of vestibular neuritis (VN) cases per quarter Significant outlier: *p=0.024, **p=0.002 CI: confidence interval

Year-season	Total number of cases examined per quarter	VN cases per quarter by examination date	Rate of VN	VN cases per quarter by date of onset
2016-4Q				2
(2017)				(0)
2018-3Q	23	0	0	1
2018-4Q	22	2	0.09	1
2019-1Q	22	0	0	1
2019-2Q	23	2	0.09	0
2019-3Q	33	1	0.03	1
2019-4Q	25	2	0.08	0
2020-1Q	20	0	0	2
2020-2Q	5	1	0.2	1
2020-3Q	16	1	0.06	2
2020-4Q	20	0	0	2
2021-1Q	20	2	0.1	0
2021-2Q	38	2	0.05	0
2021-3Q	38	5*	0.13	5**
2021-4Q	37	3	0.08	2
2022-1Q	36	2	0.06	1
Average		1.53	0.06	1.31
95% CI		(0.87–2.20)	(0.04–0.09)	(0.72–1.91)

**Table 2:** Case series of patients with VN MVS: maximum velocity of the slow phase of caloric nystagmus; CP: canal paresis; cVEMP: cervical vestibular evoked myogenic potential; oVEMP: ocular VEMP; MRI: magnetic resonance imaging; CT: computed tomography; HT: hypertension; HL: hyperlipidemia; CI: chronic ischemia; LV: poor arterial tracing of the left vertebra; LMS: left maxillary sinusitis; NSW: nonspecific white matter lesion

#	Age (years)	Sex	Onset after 1st dose (days)	Onset after 2nd dose (days)	Number of days between onset and testing	Laterality	Caloric MVS (degree/s)		CP%	cVEMP 500Hz p13-n23 (µV)		cVEMP 1kHz p13-n23 (µV)		oVEMP 500Hz n1-p1 (µV)		oVEMP 1kHz n1-p1 (µV)		Comorbidities	MRI	CT
							Right	Left		Right	Left	Right	Left	Right	Left	Right	Left			
1	85	F	65	44	30	R	0	14.5	100%	0	0	0	0	0	2.238	0	0	HT, nephrotic syndrome	CI	CI
2	79	F	81	52	19	R	0	50.5	100%	0	152.2	0	182.6	0	0	0	0	HT, HL, HC, hyperuricemia	CI, LV	CI
3	39	M	114	93	25	R	5.1	32.5	73%	238.5	305.5	286.3	235.3	0	1.393	3.133	0	No comorbidities	Not tested	Not tested
4	71	F	69	48	88	R	9.5	25.5	46%	0	0	0	0	0	4.32	7.253	0	HT, HL	Chronic ischemia	Not tested
5	50	M	46	18	17	L	30	0	100%	0	0	121.6	100.9	1.2	2.2	1.5	0	No comorbidities	No problem	No problem
6	69	M	80	59	35	L	50	5	82%	0	0	49	0	0.88	0.9	0	0	HT, HL	CI	CI
7	59	F	139	97	97	R	3	18	71%	0	0	0	81.4	0	0	0	0	HT	LMS, NSW	No problem

## Discussion

There was a significant seasonal bias with respect to the onset of VN toward 2021-3Q compared to other seasons. All patients whose VN onset was in 2021-3Q or 2021-4Q had received a COVID-19 vaccination a few months previously, suggesting a vaccination-related onset. The peripheral vestibular function in these patients was severely impaired when evaluated by caloric testing. The impaired side determined by caloric testing did not always match the impaired side determined with VEMP testing.

No seasonal variation in VN has been reported in previous studies [26,27], and no seasonal variation was observed in this study for 2019 and 2020. The onset of VN was only biased toward 2021-3Q in this study, a period when COVID-19 vaccination was enforced on a large scale, suggesting the effect of vaccination. Although the onset of dermatological symptoms associated with VZV reactivation has been reported to be about a week after vaccination [28], and the onset was two days after vaccination in a previous case report on VN [21], it is still considered possible that the onset of VN within a few months of vaccination is associated with the vaccine because the onset of facial nerve palsy after vaccination has been observed at 21 days and 30 days after receiving the first and second vaccine doses [29,30]. The COVID-19 Vaccine Adverse Event Reporting System Database in Japan includes only two cases (a 48-year-old male and a 55-year-old female) of VN out of 26,616 adverse cases in 2021. In both cases, the onset was two or three days after the vaccination and the status of both cases was evaluated as severe (grade 3). It is difficult to accurately evaluate the onset of side effects that last longer than one week unless the vaccinated and unvaccinated groups are carefully followed for a long period of time over a few months.

The CP percentage on caloric testing was 100% in most cases, suggesting severe peripheral vestibular dysfunction. Because the CP evaluated with caloric testing in the case of VN is reported to continue to be abnormal in half of the patients even after 5-10 years [31], a longer follow-up is required to determine if there is any improvement in CP in these cases.

The affected side determined by VEMPs did not necessarily match the side with CP determined by caloric testing, which was consistent with previous reports [32,33]. The comorbidities of VN cases were similar to previous reports, suggesting that the same circumstances affect the onset of VN [26,27]. These results suggest that the pathogenesis of VN after vaccination is similar to that of normal VN, whereas the absence of cold symptoms prior to the onset of VN is atypical for VN.

There are several case reports of VN following COVID-19 infection; however, the details of vestibular function examinations including caloric testing, video head impulse testing, or VEMP testing were not described in these reports [34-37] except for one report [11], and further studies evaluating the risk of VN after COVID-19 infection or VN after COVID-19 vaccination including the results of vestibular function examinations are required. A retrospective study analyzing acute peripheral vestibulopathies (APV) reported no correlation between APV and COVID-19 [38]. No statistically significant increase in VN cases in the 2020 COVID-19 pandemic season compared to 2019 was observed, consistent with this previous study. The incidence rate of VN after COVID-19 infection may vary depending on the prevalence rate of COVID-19. Although only one VN case after COVID-19 infection was observed in this study, a serological examination would be necessary to accurately detect previous COVID-19 infections.

The mechanism by which vaccines cause reactivation of the HSV is currently unknown, but one cause of HSV reactivation is presumed to be the activation of immunity [39-41], which vaccines can alter. A transient decrease in lymphocytes after vaccination is reported in one clinical trial [42], and this decrease may indicate the redistribution of lymphocytes from peripheral blood, spleen, and bone marrow to lymphatic tissue, which can be caused by the administration of cortisol or interferons [43,44], which stimulate the immune system. Most of the patients in this study were elderly, a population that is associated with a decreased functionality of the adaptive immune response compared to younger individuals [45]. The risk of VN after vaccination in young people needs to be further investigated. In addition, the serological condition in all cases of VN whose onset was between 2021-3Q and 2022-1Q in this study was clear exposure to the coronavirus spike protein, which can be a possible cause of the reactivation of the HSV.

This study has some limitations. Firstly, this was a retrospective study conducted at a single institution, which limits the quality of the data. Second, we could not accurately measure the incidence of vestibular diseases because some cases may have been treated by family physicians and not referred to the hospital where the study was conducted for further investigation. Finally, the number of cases in this study was too small to make a statistical comparison between vaccinated and non-vaccinated groups because the vaccination rate in the prefecture where the hospital in this study is located is reported to be about 80%. BNT162b2, mRNA-1273, or AZD1222 were used in this prefecture, and their respective rates were not published. The rate of vaccination in VN cases between 2021-3Q and 2022-1Q was 87.5% (7/8) in this study, which was higher than the reported vaccination rate in this prefecture, suggesting the possibility of vaccination-related VN.

Since the BNT162b2 vaccine is based on a new nucleic acid technology, it may cause side effects that have not been observed with conventional types of vaccines, and it is important to monitor any concerning symptoms in the future. Additionally, the side effects of vaccines other than RNA-based vaccines, including AZD1222, need further investigation.

## Conclusions

Cases of VN significantly increased in 2021-3Q relative to other quarters, indicating a seasonal bias relative to the timing of the COVID-19 vaccination. In many cases of VN, the onset of symptoms occurred several months after the COVID-19 vaccination, and severe semicircular canal paralysis was observed, suggesting a side effect of the vaccine. In case of vertigo attacks and vertigo symptoms after COVID-19 vaccination, a detailed vestibular function examination should be performed to accurately diagnose and treat the symptoms, and rehabilitation should be provided.

VN should be recognized as one of the side effects of the COVID-19 vaccination because it is a long-lasting side effect from which patients do not fully recover. Because of the difficulty in assessing the incidence of VN due to the retrospective nature of this study, it is important to implement a post-marketing surveillance system and continuously evaluate the safety of vaccines in order to detect events that may reduce the expected benefits of vaccination so that necessary measures to minimize the risk to the vaccinated population can be put in place.
